# Complete Isolated Ruptures of the Distal Biceps Brachii During Athletic Activity: A Systematic Review

**DOI:** 10.7759/cureus.27899

**Published:** 2022-08-11

**Authors:** Jensen G Kolaczko, Derrick M Knapik, Christopher J McMellen, Sunita R Mengers, Robert J Gillespie, James E Voos

**Affiliations:** 1 Orthopaedic Surgery, University Hospitals Cleveland Medical Center, Cleveland, USA

**Keywords:** distal biceps rupture, distal biceps, tendon rupture, bicep tendon, orthopedic sports medicine

## Abstract

Complete, isolated ruptures of the distal biceps brachii sustained during athletic activities are uncommon. A systematic review of the literature was performed to identify complete distal biceps brachii tears experienced during athletic activities to determine injury prevalence, athletic activities/mechanisms responsible for injury and return to activity timing following operative management. A total of 10 studies, comprising 16 athletes undergoing surgery for 18 cases, were identified. Injuries were predominately associated with weightlifting. Injuries were treated utilizing a single incision in 56% of cases and primary repair performed in 89% of cases. Mean time to return to activity was 4.86 ± 1.14months. Athletes undergoing surgery ≤ 10 days following injury and those undergoing primary repair returned to activity significantly quicker. Isolated tears of the distal biceps remain uncommon during athletic activities, occurring primarily during weightlifting. Return to activity timing was not significantly delayed based on surgical approach, steroid use, or athlete age.

## Introduction and background

Complete ruptures of the distal biceps brachii are rare, comprising only 3% of all biceps brachii injuries [1]. However, increased participation in contact sports, bodybuilding and weightlifting over the last two decades has resulted in an increased prevalence of distal biceps ruptures in active individuals [2-4]. Additional etiological factors reported in the literature contributing to distal ruptures include the presence of mechanical impingement, hypo-vascularity, smoking, and anabolic steroid use [5,6]. Patients sustaining ruptures treated non-operatively have reported loss of up to 40% of supination strength and variable loss of flexion strength up to 30%, along with diminished supination endurance and grip strength [7,8]. As such, for athletes and active patients desiring a return to pre-injury levels of activity, operative management is recommended [9,10].

Primary anatomic reinsertion or graft augmentation in cases of excessive tendon retraction may help prevent the loss of range of motion and strength while minimizing complications [11]. Several methods of surgical fixation as well as approaches (single versus dual incisions) have been reported [12-19]. However, return to activity timing in athletes sustaining distal biceps brachii ruptures during athletic activities treated with operative management remains unknown. 

The purpose of this study was to systematically review the literature to better understand: the prevalence of complete, isolated distal biceps brachii ruptures sustained in athletes during athletic activities; the activities responsible for injury; the impact of steroid use, interval time to surgery from injury, operative approach (single versus dual incision), and fixation method (primary repair versus graft augmentation) on outcomes and return to activity timing. Based on prior investigations, the authors hypothesized a low prevalence of injuries occurring primarily during weightlifting with no significant differences in return to activity based on steroid use, time to surgery, surgical approach or repair method [2,3,9].

## Review

A systematic review was conducted according to the Preferred Reporting Items for Systematic Reviews and Meta-Analyses (PRISMA) guidelines using a PRISMA checklist [20]. All literature pertaining to individuals sustaining complete, isolated distal bicep brachii tendon ruptures during athletic activities published between January 1954 and December 2018 was identified. The authors considered an athletic activity as one in which the athlete was either in competition or participating in strenuous exercise to improve physical strength or endurance. Two authors, both resident physicians, independently conducted a literature search in October 2018 using the following databases: Biosis Previews, SPORTdiscus, PEDRO and EMBASE. Each search included the following terms: sport AND distal bicep brachii AND tendon AND rupture AND tear.

The inclusion criteria consisted of English language or articles with English translations, human subjects sustaining complete, isolated distal biceps tendon ruptures during athletic activity treated with operative management, reported surgical approach, along with activity and mechanism of injury, as well as return to activity timing. Exclusion criteria were: non-English language articles, athletes sustaining partial tears of the distal biceps brachii, athletes sustaining tears with concomitant injuries to the ipsilateral upper extremity, tears occurring during non-athletic events (activities not meeting the definition of an athletic activity), tears without reported, complete tears requiring operative intervention secondary to initial failed non-operative management, and studies not reporting injury treatment or return to activity timing. 

**Figure 1:**
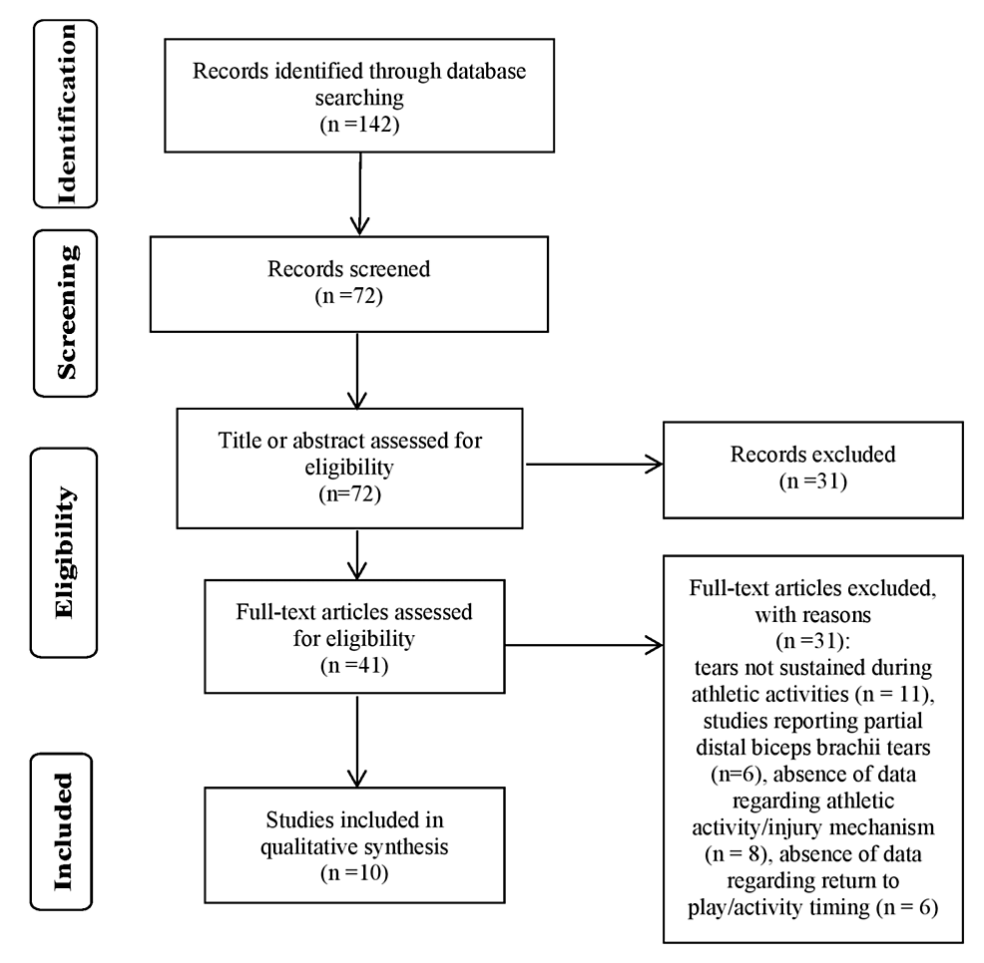
Preferred Reporting Items for Systematic Reviews and Meta-Analyses (PRISMA) flowchart of study

Following the two independent authors' search of the literature, a total of 142 citations were identified. The search process is shown in the flow diagram (Figure 1). Following title and abstract evaluation, a total of 41 articles were selected for further evaluation. Of these studies, 31 studies were excluded due to: tears not sustained during athletic activities (n = 11), studies reporting partial distal biceps brachii tears (n = 6), absence of data regarding athletic activity/injury mechanism (n = 8), or absence of data regarding return to play/activity timing (n = 6). Following application of the inclusion/exclusion criteria, a total of 10 studies were identified for analysis. To ensure that all available studies were identified, references cited in the included articles were cross-referenced for inclusion if they were overlooked during the initial search. 

Statistical analysis was used to compare mean time to return to activity in athletes based on surgical approach (single versus dual incision), repair type (primary repair versus graft augmentation), history of steroid abuse, patient age (≤ 29 versus ≥ 30 years of age at the time of injury), and interval time to surgery. Surgical timing was separated into athletes treated ≤ 10 days from injury versus those treated in delayed fashion, > 10 days following injury as defined by Kelly et al. who reported an increased rate of post-operative complications in patients treated greater than 10 days following injury [21]. Student’s t-test was performed with a p-value of <0.05 to determine statistical significance. All statistical analyses were performed using SPSS software (Version 23, IBM, Armonk, NY, USA). 

A total of 10 studies, comprising 16 athletes undergoing surgery for 18 cases of complete, isolated ruptures of the distal biceps tendon occurring during athletic activity, were identified (Table 1). The mean age of athletes at the time of injury was 30.6 ± 12.9 years with 94% (n = 15 of 16 athletes) being male. Mean final follow-up time following surgery was 26 ± 20.5 months. Two athletes, one of whom was taking high-dose anabolic steroids, sustained bilateral simultaneous distal biceps tears during weightlifting and required repair [22,23].

**Table 1:** Summary of studies included in review Legend:  M, male athlete; F, female athlete; IT, iliotibial; ROM, range of motion; UC, ulcerative colitis; R, right arm; L, left arm; PMHx: past medical history; NR, not recorded; *, bilateral ruptures; †, 5 degree loss of flexion, 10 degree loss of extension.

Study	Journal (year)	Level of Evidence	Sex/ Age	Activity	Approach	Primary v. Graft Repair	Injury to Surgery Interval (days)	Complications	Steroid Use	Return to Activity (months)
Hovelius et al. [24]	Acta Orthop Scand. (1977)	4	M/53	Handball	Single Incision	Primary	NR	None	NR	6
Baker and Bierwagen [7]	JBJS (1985)	4	M/43	Weightlifting	Dual Incision	Primary	1	Myositis Ossificans w/ROM loss†	NR	4
Louis et al. [25]	Am J Sports Med (1986)	5	M/22	Weightlifting	Single Incision	Primary	1	None	NR	3
Visuri et al. [23]	Med. Sci. Sports Exerc. (1993)	5	M/23*	Weightlifting	Dual Incision	Primary	NR	None	Yes	2.5
				Boxing	Dual Incision	Primary	NR	None	Yes	3
Williams et al. [10]	Phys Sportsmed (1996)	5	M/25	Snowboarding	Dual Incision	Primary	6	NR	No	6
Thompson [26]	J Athl Train. (1998)	5	M/21	American Football	Dual Incision	Graft (IT Band)	60	None	No	6
Toczylowski et al. [27]	J. Shoulder Elbow Surg (2002)	5	F/58	Ice Skating	Dual Incision	Primary	10	None	NR	4
Rokito and Iofin [22]	Bull NYU Hosp Jt Dis. (2008)	5	M/51*	Weightlifting	Dual Incision	(R) Primary (L) Achilles Graft	(R) 42 (L) 140	NR	No	6
Gupta et al. [28]	Indian J Orthop (2012)	4	M/24	Weightlifting	Single Incision	Primary	3	None	NR	5
			M/26	Weightlifting	Single Incision	Primary	1	None	NR	5
			M/21	Boxing	Single Incision	Primary	6	None	NR	5
			M/28	Weightlifting	Single Incision	Primary	7	None	NR	5
			M/29	Weightlifting	Single Incision	Primary	10	None	NR	5
			M/23	Kabaddi	Single Incision	Primary	7	None	NR	5
			M/26	Wrestling	Single Incision	Primary	6	None	NR	5
Ding et al. [29]	J Pediatr Orthop B. (2016)	5	M/17	Weightlifting	Single Incision	Primary	10	None	Yes, Chronic (PMHx: asthma, UC, hypothyroid)	6

Weightlifting was the most common athletic activity responsible for injury, reported in 56% (n = 9 of 16) of athletes [7,22-26], followed by boxing (13%, n = 2 of 16) [23,28]. Three percent of all biceps injuries are distal biceps tears with an estimated incidence of 2.55 to 5.25 per 100,000 per year with the vast majority of other injuries involving the proximal long head of the biceps tendon [2]. Injuries were also reported in athletes participating in American football [26], ice skating [27], handball [24], snowboarding [10], Kabaddi [28], and wrestling [28]. Kabaddi is a high-energy contact sport popular throughout India and South Asia.

The mean time from injury to surgery was 20.7 ± 36.9 days, while time to surgery was not recorded in three cases (n = 2 athletes) [23,24]. Distal biceps tendon repair was performed using a single incision technique in 56% (n = 10 of 18) of procedures. Primary repair was undertaken in 89% (n = 16 of 18) of cases, while autograft harvested from the iliotibial band [26] and Achilles tendon [22] was utilized in two cases due to the extent of tendon retraction secondary to delay from time of injury to initial presentation. Notably, the cases requiring autograft occurred 60 and 140 days, respectively, after injury compared to the cases utilizing primary repair, in which surgery occurred within 10 days of injury. No intra-operative complications were reported, while only a single (6%, n = 1 of 18) post-surgical complication was documented secondary to development of myositis ossificans after a two-incision approach with subsequent limitation in range of motion when compared to the non-operative elbow at final follow up [7]. Almost 13% (n = 2 of 16) of athletes reported a history of steroid use prior to rupture, with one athlete sustaining bilateral ruptures reporting long-term anabolic-androgenic steroid abuse beginning six years before rupture [23]. Meanwhile, one athlete reported long-term systemic steroid use secondary to medical necessity due to asthma, hypothyroidism and ulcerative colitis [29]. Three athletes denied steroid use [10,22,27] while explicit acknowledgment or denial of steroid use was not provided in five studies [7,24,25,27,28].

Mean overall time to return to activity was 4.86 ± 1.14 months (range, 2.5 to 6 months) following operative fixation. No significant difference in return to activity was appreciated in athletes treated with single (5.0 ± 0.82 months) compared to dual (4.69 ± 1.49 months) incisions (p=0.60) or in athletes with a history of steroid use (3.83 ± 1.89 months) compared to those without (6.0 ± 0 months) (p=0.19). Athletes undergoing primary repair returned to activity significantly quicker (4.91 ± 1.35 months) when compared to those treated with graft augmentation (6.0 ± 0 months) (p=0.0004), which could in part be a surrogate for delayed fixation leading to a prolonged recovery. There was no difference in post-operative protocols reported for primary repair compared to those treated with graft augmentation. Age at the time of injury did not have a significant impact on return to activity timing in patients ≤ 29 years of age (4.73 ± 1.17 months) versus those ≥ 30 years of age (5.2 ± 1.1 months) (p=0.45). Athletes undergoing surgery ≤ 10 days following injury returned to activity significantly earlier when compared to athletes treated in delayed fashion > 10 days from injury (4.83 ± 0.83 months versus 6 ± 0 months, respectively; p=0.0005).

The principal findings from this investigation were that in the 16 identified athletes sustaining a total of 18 complete, isolated distal biceps ruptures during athletic activity, 94% were males while weightlifting was responsible for 56% of injuries. During operative repair, a single incision approach was performed in 56% of cases with primary repair performed in 89% of cases. Mean overall time for return to activity following operative repair was 4.86 ± 1.14 months. Time to return to play was not significantly affected in athletes with a history of steroid use, those undergoing surgery using a single versus dual incision, or age at time of surgery. However, those subjects undergoing surgery ≤ 10 days following injury and those treated with primary repair returned to activity significantly quicker.

Differences in anatomic and biomechanical properties effectively place males at an increased risk of tendon rupture. Results from this study found that 94% of athletes sustaining distal biceps brachii ruptures during athletic activity were male, reflective of the predominance of previously reported distal biceps brachii injuries in males when compared to females [2,19,30,31]. To date, only four reported cases of females sustaining distal biceps tendon ruptures are present in the literature [3,27,32]. Toczylowski et al. postulated that this sexual disparity may be due to the greater cross-sectional area (CSA) and increased percentage of lean muscle mass in males when compared to females, capable of generating higher forces, and increasing the risk for rupture [27]. Moreover, males possess significantly larger muscle fibers in the biceps brachii muscle compared to females, with females capable of generating only 52%-60% of the flexion strength seen in males [33,34]. As such, in conjunction with biomechanical and anatomic differences, the increased prevalence of males participating in weight training may place males at high risk for ruptures when compared to females, however further studies examining weightlifting as a potential causative factor are warranted.

Single incision was utilized more frequently compared to a dual incision approach in the athletes evaluated in this study. While the single incision approach allows for the utilization of several types of fixation methods [35], the dual incision primarily utilizes suture fixation via bone tunnels [36] to obtain adequate fixation, as observed in the athletes reported in this review treated using the dual incision approach [7,10,22,23,26,27]. Classically, single incisions have been associated with a higher incidence of nerve injury, specifically lateral antebrachial cutaneous nerve (LACN) palsy secondary to forceful traction [37,38]. Dunphy et al. corroborated this increased incidence of LACN palsy using the single incision approach in their review of 784 surgical repairs, reporting an incidence of 24.4% in patients treated using a single incision versus only 4.1% treated with a dual incision [30]. In contrast, the dual incision approach is associated with a higher rate of heterotopic ossificans and radial-ulnar synostosis. In their meta-analysis, Rantanen et al. found that 71.4% of patients undergoing fixation utilizing the dual incision approach developed evidence of radio-ulnar synostosis post-operatively [31], believed to be secondary to disruption of the ulnar periosteum and intraosseous membrane, along with bone debris from the drilling of bone tunnels, stimulating bone formation [39,40]. These complications led to the popularization of the modified Boyd-Anderson (Mayo) approach utilizing a muscle spitting dissection plane dorsally, avoiding exposure of the ulna and decreasing the likelihood of heterotopic ossification and synostosis [8,41]. In their retrospective review analyzing 74 primary repairs utilizing the Mayo approach, Kelly et al. confirmed the safety of the Mayo approach, reporting no cases of radio-ulnar synostosis and only four cases of non-rotation limiting heterotopic ossification [21]. Based on the studies included in this review, surgical approach did not increase the risk of complications or impact return to activity timing during repair of complete, isolated distal biceps brachii tendon ruptures sustained during athletic activity.

Significant differences in return to activity timing were appreciated based on surgical timing from initial injury. In cases of delayed presentation following injury, augmentation may be necessary due to proximal retraction of the tendon with associated adhesion formation, commonly reported in injuries sustained six to eight or more weeks prior to surgery [21,42]. In this review, the two athletes undergoing graft augmentation initially presented in delayed fashion, at greater than six weeks following injury [22,27]. While augmentation utilizing fascia lata, semitendinosus, Achilles tendon, gracillis, tibialis anterior, and the flexor carpi radialis graft have been described in the literature [22,23,43-45], only Phadnis et al. have described return to activity following graft augmentation [46]. The authors reported full return to activity, including rugby, soccer, American football, cricket, and boxing in 100% (n = 21 of 21) of cases treated with Achilles tendon allograft in patients undergoing fixation at a mean of 25 months following injury, however, return to play timing was not provided [46].

Similarly, the increased time to return to activity in patients treated in delayed fashion (> 10 days) may be attributed to the use of graft augmentation in the two patients undergoing delayed fixation [22,26]. However, due to the small number of athletes treated with graft augmentation and in delayed fashion in this review, no definitive evidence regarding optimal repair method combined with surgical timing can be truly extrapolated from the included studies. As such, despite a statistical difference in return to activity if surgical repair was pursued within 10 days from injury and the use of a primary repair was utilized, the clinical relevance of these findings is unknown and additional prospective studies evaluating these factors are necessary to determine the true impact of repair type and surgical timing on return to activity timing. 

Steroid use has been identified as a risk factor for distal biceps brachii tendon ruptures [47]. Steroids have been shown to have an anabolic effect on the contractile proteins, increasing muscular strength but also leading to dysplastic changes within the collagen. These dysplastic changes decrease the ability of the tendon to stretch and absorb force, increasing the likelihood of rupture, especially during eccentric loading [11,48,49]. Tendons have also been shown to become stiffer and unable to withstand the high loads placed by the contracting muscle [48]. While the use of steroids was not found to impact return to play time in the current study, the small sample size of athletes acknowledging steroid use prohibits the authors from determining the true impact of steroid use on outcomes following complete, isolated distal biceps brachii tendon ruptures.

Aging has also been shown to produce deleterious effects on the structural integrity of the distal biceps brachii tendon. With increasing age, cross-linking of collagen fibers increases tendon stiffness while decreasing compliance [50]. This decrease in elasticity lowers the ability of the tendon to resist tensile load, predisposing the tendon to rupture [27]. In their study of 302 biceps brachii tendon ruptures (mean patient age, 63 ± 8.0 years), Kannus and Jorza reported the most consistent finding associated with tearing was the presence of degenerative tendinopathy [50]. While not significant in this study, age has been shown to delay return to activity in older patients, as Cohen et al. reported a mean return to play time of seven months in 58 patients undergoing repair at a mean age of 53 years [14]. 

This study was not without limitations. Due to the inherent heterogeneity of the articles included with regards to functional outcome scores, strength testing, range of motion measurements and subjective outcome scores, no reliable statistical analysis could be performed to assess these variables. Furthermore, due to the strict inclusion/exclusion criteria utilized, the small sample size obtained for athletes treated using graft augmentation, in delayed fashion, or those reporting a history of steroid use limits the clinical applicability of the data extrapolated from the included studies. The generalizability of these outcomes to the general population sustaining complete, isolated distal biceps ruptures requiring operative repair secondary to non-athletic related trauma are unknown and cannot be inferred based on the population analyzed in this review. Additionally, the finding of delay in return to sport with the use of graft augmentation compared to primary repair may be confounded by the delayed presentation of these patients and additional studies with larger sample sizes will be necessary to confirm this finding. 

## Conclusions

In conclusion, ruptures of the distal biceps brachii tendon during athletic activity remain infrequent with the majority of athletic activity-related tears occurring during weightlifting in males, treated using a single incision with primary repair. No significant difference in return to play timing was appreciated based on surgical approach, steroid use or patient age at the time of surgery. While return to activity timing was significantly increased in athletes treated with graft augmentation and in those who had surgical repair > 10 days from injury, the clinical relevance of these findings is unknown given the small sample size of athletes identified. As such, future studies evaluating prospective outcomes based on steroid use, repair techniques and timing are necessary to determine the true impact of these variables in active patients to ensure quick and safe return to pre-injury levels following complete, isolated distal biceps brachii ruptures. 
